# Role of Emerin in regulating fibroblast differentiation and migration at the substrate of stiffness coupled topology: Emerin regulates fibroblast differentiation and migration

**DOI:** 10.3724/abbs.2024094

**Published:** 2024-07-08

**Authors:** Tiantian Yang, Li Wang, Haiyang Ma, Kailun Li, Yajing Wang, Wenjie Tang, Zichen Wang, Meiwen An, Xiang Gao, Ludan Xu, Yunyun Guo, Jiqiang Guo, Yong Liu, Hugen Wang, Yang Liu, Quanyou Zhang

**Affiliations:** 1 College of Biomedical Engineering Taiyuan University of Technology Taiyuan 030024 China; 2 Trauma Center Trauma Orthopaedics ZhouKou Orthopaedic Hospital Zhoukou 466000 China; 3 Shanxi Bethune Hospital the Third Hospital of Shanxi Medical University Taiyuan 030053 China; 4 Dermatology Department Shanxi Bethune Hospital Shanxi Academy of Medical Sciences Taiyuan 030032 China; 5 Orthopaedics department the First People’s Hospital of Jinzhong Jinzhong 030600 China; 6 Department of Nuclear Medicine the First Hospital of Shanxi Medical University Taiyuan 030012 China; 7 Department of Orthopaedics Shanxi Medical University Taiyuan 030001 China

**Keywords:** hyperplastic scar, matrix microenvironment, nuclear skeleton protein, cell differentiation, cell migration

## Abstract

In hypertrophic scars, the differentiation and migration of fibroblasts are influenced by the extracellular matrix microenvironment, which includes factors such as stiffness, restraint, and tensile force. These mechanical stresses incite alterations in cell behavior, accompanied by cytoskeletal protein reorganization. However, the role of nucleo-skeletal proteins in this context remains underexplored. In this study, we use a polyacrylamide hydrogel (PAA) to simulate the mechanical stress experienced by cells in scar tissue and investigate the impact of Emerin on cell behavior. We utilize atomic force microscopy (AFM) and RNA interference technology to analyze cell differentiation, migration, and stiffness. Our findings reveal that rigid substrates and cellular restriction elevate Emerin expression and diminish differentiation. Conversely, reducing Emerin expression leads to attenuated cell differentiation, where stiffness and constraining factors exert no notable influence. Furthermore, a softening of cells and an enhanced migration rate are also markedly observed. These observations indicate that variations in nuclear skeletal proteins, prompted by diverse matrix microenvironments, play a pivotal role in the pathogenesis of hypertrophic scars (HSs). This research offers novel insights and a reference point for understanding scar fibrosis formation mechanisms and preventing fibrosis.

## Introduction

Changes in the extracellular matrix are a distinguishing feature of hypertrophic scars (HSs) compared to normal skin [1]. During the scarring phase, fibroblasts facilitate wound repair through migration, differentiation, and the synthesis, secretion, and deposition of the extracellular matrix [ 2, 3] . This process results in excessive collagen deposition, leading to tissue hardening. The physical morphology of collagen fibers shifts from a parallel arrangement in normal wound skin to a disordered, cross arrangement in scars [4]. This abnormal extracellular matrix in scar tissue generates various mechanical stimuli for cells, leading to significant cellular changes. Numerous studies have reported that substrate stiffness significantly impacts cell behavior, influencing cell spread size [5], cytoskeleton arrangement [6], and the degree of cellular fibrosis [7]. A notable effect of topology on cells is the alteration of their morphology and orientation [8]. In this context, grooved substrates exert a more pronounced influence on cell orientation than other topologies [9]. Cells appear rounder and smaller when the topology is submicron in size [10], whereas they exhibit enhanced spreading and orientation on micrometer-sized substrates [11]. The extracellular matrix constitutes a complex regulatory environment in which multiple mechanical stimuli may jointly influence cellular behavior. While significant research has focused on the independent roles of topology and stiffness in HS, the impact of their combined effects on cell behavior warrants further detailed investigation.

Emerin, a member of the laminus-associated protein family, is predominantly expressed at the inner nuclear envelope and plays diverse roles in polar cell organization, nuclear stability, chromatin binding, gene expression, cell signaling, and mechanical transduction. Emerin-deficient mouse embryonic fibroblasts exhibit abnormal nuclear morphology characterized by increased nuclear deformability, diminished resistance to mechanical stress, and impaired mechanotransduction [12]. Emerin is involved in the regulation of the front-rear polarity of the nucleus [13]. In human myotonic dystrophy cells, Emerin facilitates the transmission of mechanical forces from the cytoskeleton to the nucleoskeleton, significantly influencing cell division, cytoskeletal remodeling, and organelle localization [14]. Additionally, in human myofibroblasts with atrial cardiac defects, a reduction in the expression of the Emerin domain ∆K37 has been observed, leading to deficiencies in actin stress fiber production [15]. Recent studies have also explored the role of Emerin in wound healing, where it stimulates SRF-Mkl1-dependent gene activity in a substrate stiffness-dependent manner, thus enhancing myofibroblast differentiation during the healing process [16]. Emerin is increasingly recognized as a key regulator of cellular behavior. However, its biological function in fibroblast migration and differentiation remains unclear.

In this study, we hypothesized that (i) stiffness-coupled topology influences fibroblast morphology, migration, and differentiation; (ii) erastin affects these fibroblast characteristics on the substrate; and (iii) changes in fibroblast stiffness impact cell migration. We designed PAAs with various stiffnesses and topological properties to mimic different matrix microenvironments. This approach aims to elucidate the formation mechanisms of HS and provide novel insights and targets for its effective prevention and control, offering both theoretical significance and practical value.

## Materials and Methods

### Preparation and processing of stiffness-coupled topology substrates

Three silicon plate topologies were used: parallel grooves, crossed 90° grooves (with a restricted groove length of 200 μm and a 90° angle between restricted and unrestricted grooves), and crossed 45° grooves (with a restricted groove length of 200 μm and a 45° angle between restricted and unrestricted grooves). All topologies feature a ridge and groove width of 15 μm and a depth of 10 μm. Hydrogel substrates were prepared following the method outlined previously [ 17– 19] . Specifically, acrylamide (AM; Amresco, Washington, USA), bisacrylamide (Bis; Sigma-Aldrich, St Louis, USA), and water were mixed according to the solution ratios presented in Table 1. To this mixture, 1/200 (v/v) of 10% ammonium persulfate (17874; Thermo Fisher Scientific, Waltham, USA) as a curing agent and 1/2000 (v/v) of the accelerator N,N,N *′*,N *′*-tetramethylethylenediamine (110-18-9; Aladdin, Shanghai, China) were added. The prepared hydrogel solution was poured into a 6-cm Petri dish, covered with a photolithography plate, and left to set for 30 min at room temperature. The cured hydrogel was then removed and immersed in hydroxyethylpiperazine ethanesulfonic acid (HEPES; Merck, Darmstadt, Germany) ionic washing solution for 48 h.

**Table:** **Table 1** Reagent ratios for PAA preparation

40% AM (mL)	2% Bis (mL)	Water (mL)
2.5	0.36	7.14
2.5	3.0	4.5

### Hydrogel surface treatment

The hydrogel, 1 mm in thickness, was cut into 1 cm diameter circles using a mold. Two hundred microliters of sulfo-SANPAH (22589; Thermo Fisher Scientific) at a concentration of 0.2 mg/mL was applied to each gel surface. The surface was then exposed to UV light for 30 min using a UV lamp (Yi Peng; Shanghai, China), and the residual cross-linker was removed with HEPES and phosphate buffer solution (PBS, SC106-01; Sevenbio, Beijing, China). Subsequently, 500 μL of rat tail type I collagen (Solarbio, Beijing, China) at a concentration of 200 μg/mL was added to the surface of each hydrogel and incubated for 8-12 h in a cell culture incubator (Thermo Fisher Scientific).

### Measurement of the hydrogel elastic modulus

An Instron 5544 dynamic and static fatigue tester (Instron, Massachusetts, USA) was used for compression testing. The needle radius ( *R*) was 0.5 mm, with a compression speed of 1 mm/min and a compression thickness of 1 mm. Measurements were taken at five different points on the gel block, and each point was compressed five times ( *n*=5) to obtain displacement and stress data. The elastic modulus of the substrate was calculated using Equation (1), where E is the modulus of elasticity, δ is the strain, F is the stress, and R is the needle radius, with *ν*=0.5 and 2 *R*= 1 mm.

**Table:** 

$$\begin{array}{cr} E=F\times \left(1-v^{2}\right)/2R\delta & \text{(1)} \end{array}$$

### Cell extraction and culture

Human skin fibroblasts (HSF) were isolated using the collagenase digestion method. Foreskin tissue was obtained following circumcision at Shanxi Bethune Hospital from a healthy individual. All human cell studies were conducted with ethical approval from the Institutional Review Board of Shanxi Bethune Hospital. The tissue was disinfected with povidone-iodine (Hynaut, Qingdao, China) and PBS containing a 1% penicillin-streptomycin mixture (P1400; Solarbio), followed by the removal of subcutaneous tissue. The tissue was then incubated overnight at 4°C in 0.25% Dispase II solution (D6430; Solarbio) to separate the epidermis from the dermis. The dermal tissue was cut into 1-mm³ pieces and digested in 0.2% type II collagenase solution (C8150; Solarbio) at 37°C for 3-5 h, followed by centrifugation at 225 *g* for 5 min. The cells were cultured in DMEM (SH30022.01; HyClone, Logan, USA) supplemented with 10% fetal bovine serum (10099141; Gibco, Carlsbad, USA) and 1% penicillin-streptomycin mixture. Culturing was conducted in a 37°C incubator with 5% CO _2_, and the medium was changed every 48 h. Experiments were performed with cells at the 2nd to 4th passages, and subsequent experiments commenced when confluency in the culture flasks reached 70%.

### Cell seeding and live imaging

Once the cells reached 70% confluence, they were trypsinized (C8150; Solarbio) and seeded onto hydrogel substrates at a density of 5×10 ^4^ cells/mL. The live cell continuous photomicrograph microscope system Cystation5 (Thermo Fisher Scientific) was set to 37°C and 5% CO _2_. A 12-well plate with hydrogel was then placed into the instrument. A field of view with an appropriate cell number was selected, with three replicate samples for each substrate type. The automatic time-lapse imaging program was initiated with an imaging interval of 20 min and a duration of 4 h. The images obtained were processed and analyzed using Image J software (NIH, Bethesda, USA), and the length, width, and area of the cells were quantified. For each group, 20 cells were measured from three independent replicate experiments.

### Measurement of the cytoplasmic elastic modulus by atomic force microscopy (AFM)

After 4 h of culture on the hydrogel substrate, the hydrogel was transferred to a 100-mm dish containing PBS. The cytoplasmic elastic modulus was measured using an atomic force microscope (Bruker, Billerica, USA) combined with an inverted phase contrast microscope (IX-70; Olympus, Tokyo, Japan) on live cells in culture dishes. The indentation test utilized a rigid spherical indentation cantilever beam with a diameter of 2 μm (the spring constant was 0.05 N/m, MLCT-O10; Bruker). The force was controlled at 1 nN, and the probe measurement depth was set to 2 μm. The cytoplasmic elastic modulus was determined by fitting the force-distance curve using Hertz’s model and calculated using Equation (2),

**Table:** 

$$\begin{array}{cr} \text{F}\text{=}\frac{2}{\text{π}}\frac{\text{E}}{{\text{1-}\text{v}}^{2}}\tan\left(\text{α}\right){\text{δ}}^{2} & \text{(2)} \end{array}$$

where *E* is the desired surface modulus of elasticity, *δ* is the depth of the incision, *F* is the applied force, *α* is the probe half-tension angle of 18°, and *ν* is the Poisson’s ratio, which is assumed to be 0.5.

### Immunofluorescence staining

Emerin-treated cells were seeded on hydrogels and incubated for 4 h. The cells were fixed with methanol (P0098; Beyotime, Shanghai, China) for 5 min at room temperature and then washed with PBS. The cells were permeabilized using an immunofluorescence permeabilization solution (P0096; Beyotime) for 10 min, followed by another wash with PBS. The cells were blocked with a 1% BSA sealing solution for 30 min. They were then incubated with the primary antibody against Emerin (1:500, ab40688; Abcam, Cambridge, UK) for 1 h at room temperature and washed with PBS. The cells were incubated with Alexa Fluor 488-conjugated goat anti-rabbit secondary antibody (1:400, ab6717; Abcam) for 1 h at room temperature, followed by another wash with PBS. The nuclei were stained with 4′,6-diamidino-2-phenylindole (DAPI) (C0065; Solarbio) for 30 min at room temperature and then washed with PBS.

For α-smooth muscle actin (α-SMA) staining, cells on hydrogels were fixed using an immunofluorescence fixative (P0098; Beyotime) for 20 min at room temperature and washed with PBS. The cells were permeabilized for 10 min, followed by washing with PBS. The cells were blocked with an immunofluorescence sealing solution (P00102; Beyotime) for 1 h, and then incubated with primary anti-α-SMA antibody (bsm-52396R; Bioss, Beijing, China) for 90 min at room temperature, followed by wash with PBS. The cells were then incubated with Alexa Fluor 488-conjugated goat anti-rabbit secondary antibody (1:400, ab6717; Abcam) for 1 h at room temperature, followed by DAPI staining of the nuclei for 30 min. Cell imaging was performed using a Cytation 5 microscope (Bio Tek, Winooski, USA), and the images were processed and analyzed using ImageJ software (NIH).

### Transfection with small interfering RNA (siRNA)

Prior to transfection, HSF cells were seeded in 24-well plates at a density of 1×10 ^5^ cells/mL, with approximately 5×10 ^4^ cells per well. After 8-12 h, when HSF confluency reached 60%-70%, Emerin siRNA (gene ID: 2010, cat# NM_000117) and control siRNA (1027281) were transfected into the cells using Lipofectamine RNAiMAX (13778075; Thermo Fisher Scientific) according to the manufacturer’s protocol. The medium was changed 6 h posttransfection. After 48 h, gene and protein alterations were assessed. These siRNA sequences were synthesized by Sangyo Bioengineering (Shanghai, China).

### Quantitative real-time polymerase chain reaction (qRT-PCR)

Total RNA from different HSF groups was extracted using the Eaststep Super Total RNA Extraction Kit (LS1040; Promega, Beijing, China). Reverse transcription was carried out using the Mir-X miRNA First-Strand Synthesis Kit (638313; Takara, Shiga, Japan), with gDNA removal and cDNA synthesis performed using the PrimeScript™ RT kit (RR047A; Takara). cDNA was amplified using TB Green Premix (638319; Takara). Quantitative real-time PCR was performed using the StepOnePlus™ real-time PCR system (Thermo Fisher Scientific). The results were analyzed using the 2 ^‒ΔΔCT^ method with *GAPDH* as the internal reference for mRNA. The experiment was conducted in triplicate. All oligonucleotide sequences are listed in Table 2.

**Table:** **Table 2** Sequences of primers for real-time quantitative PCR

Gene	Forward primer (5′→3′)	Reverse primer (5′→3′)
*GAPDH*	CAAGGGCATCCTGGGCTACACT	CTCTCTCTTCCTCTTGTGCTCTTGC
*Emerin*	TCCGCCGCCTCCTCTTATAGC	GTCATTGTAGCCCTTGCTCTGGTAG

### Western blot analysis

Total protein was extracted using RIPA lysis buffer (SW104-02; Sevenbio, Beijing, China), and its concentration was determined with a BCA protein assay kit (SW101-02; Sevenbio). Approximately 20 μg of protein sample was separated on a 10% SDS-PAGE gel (SW109-01; Sevenbio). Following electrophoresis, proteins were transferred to a PVDF membrane (Immobilon, Darmstadt, Germany). The membrane was blocked with a blocking solution (SW127-02; Sevenbio) for 1.5 h. Next, the membrane was incubated with a primary antibody (1:500, ab40688; Abcam, Cambridge, UK) or anti-GAPDH (1:2000, AG019; Beyotime, Shanghai, China) overnight at 4°C, followed by incubation with HRP-conjugated anti-rabbit IgG H&L secondary antibodies (Abcam) for 1.5 h. Solutions A and B from the ultrasensitive ECL chemiluminescence kit (SW134-01; Sevenbio) were mixed and applied to the membrane, which was then visualized using a gel imager. Image acquisition was conducted with a Tanon 6400 automated chemiluminescence image analysis system (Tanon, Shanghai, China). The resulting bands were processed and quantified using Image J software using GAPDH as an internal reference.

### Statistical analysis

Statistical analysis was conducted using GraphPad Prism 8.0 (GraphPad Software, La Jolla, USA). Data are expressed as the mean±standard deviation (SD) for the indicated number of experiments. Two-way ANOVA and Student’s *t* test were used to assess statistical significance, with the Bonferroni correction as the post hoc method for ANOVA. Each experiment was independently repeated three times. All values are reported as the means with a 95% confidence interval. *P*<0.05 indicated significant difference.

## Results

### Preparation of stiffness-coupled topological substrates

**Figure 1:**
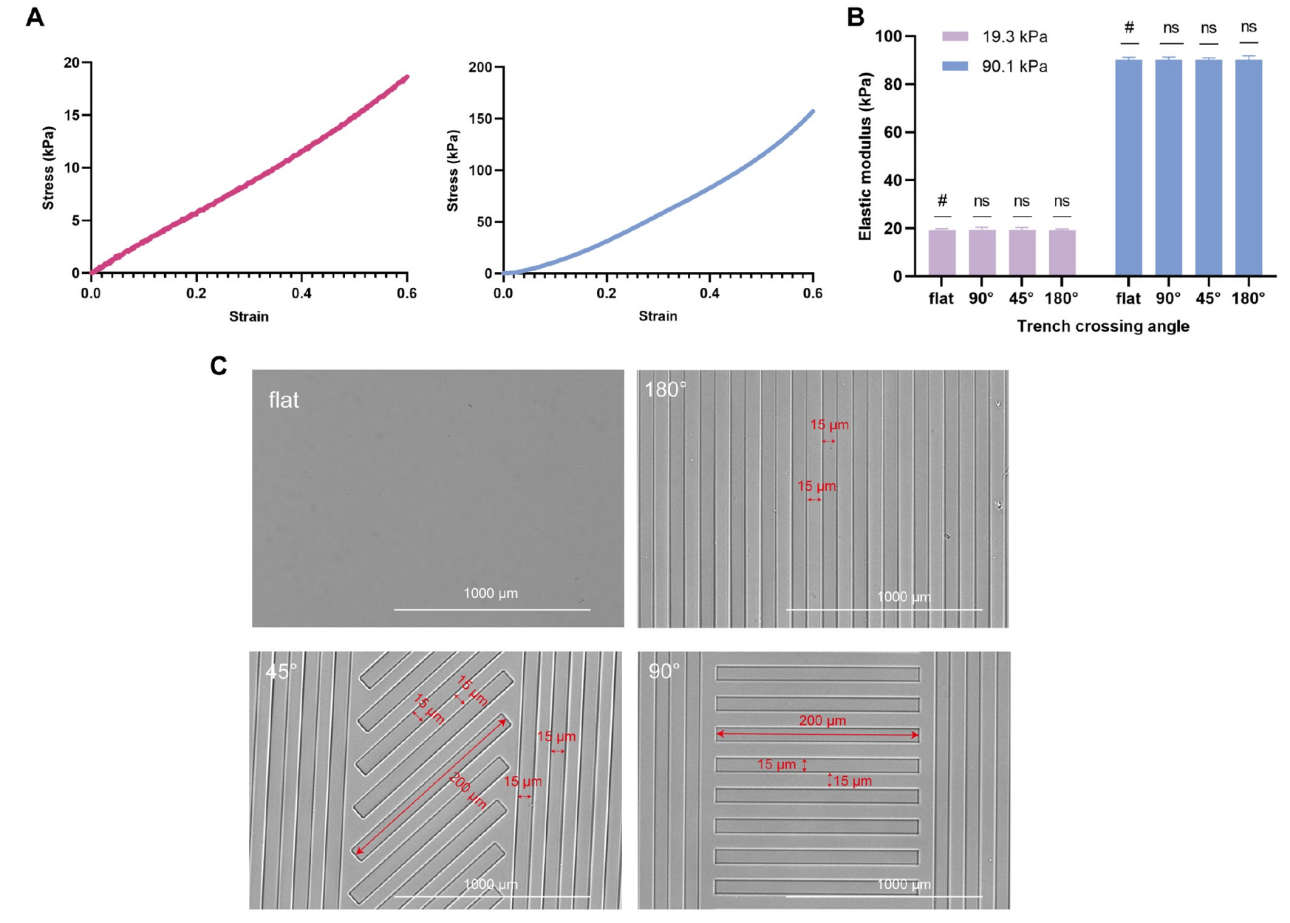
Figure 1 Morphological and physical characterization of stiffness-coupled topology substrates (A) Stress-strain curves of hydrogels with two different stiffnesses. (B) Elastic modulus measurements of hydrogels with various topologies, demonstrating no significant impact of topology on the elastic modulus. (C) Representative topography image of a 19.3 kPa substrate. Statistical analysis between groups was conducted using a t test.

According to prior research on the extracellular matrix stiffness of traumatized and scarred skin [20], we engineered substrates replicating the stiffness of both normal and scarred skin using elasticity-tunable polyacrylamide hydrogels. The disorganized and crossed collagen fiber arrangements in scar tissue were simulated using crossed 45° and 90° grooves, while the parallel collagen fiber arrangement in normal traumatized skin was mimicked using 180° parallel grooves. The stiffness of the hydrogels was quantified, with Figure 1A illustrating the stress-strain curves of two planar hydrogels exhibiting elastic moduli of 19.3±1.09 kPa and 90.1±1.58 kPa. To confirm whether the hydrogel surface topology influences the Young’s modulus, mechanical testing was conducted on hydrogels with different topologies, revealing no significant impact on stiffness ( Figure 1B, *P*<0.05). The surface morphology of the hydrogels was examined using the Cytation 5 microscope ( Figure 1C), confirming the successful creation of a topography (depth×width×spacing= 10 μm×15 μm×15 μm) on the 19.3 kPa hydrogel.

### Influence of stiffness-coupled topology substrates on the HSF spreading morphology

**Figure 2:**
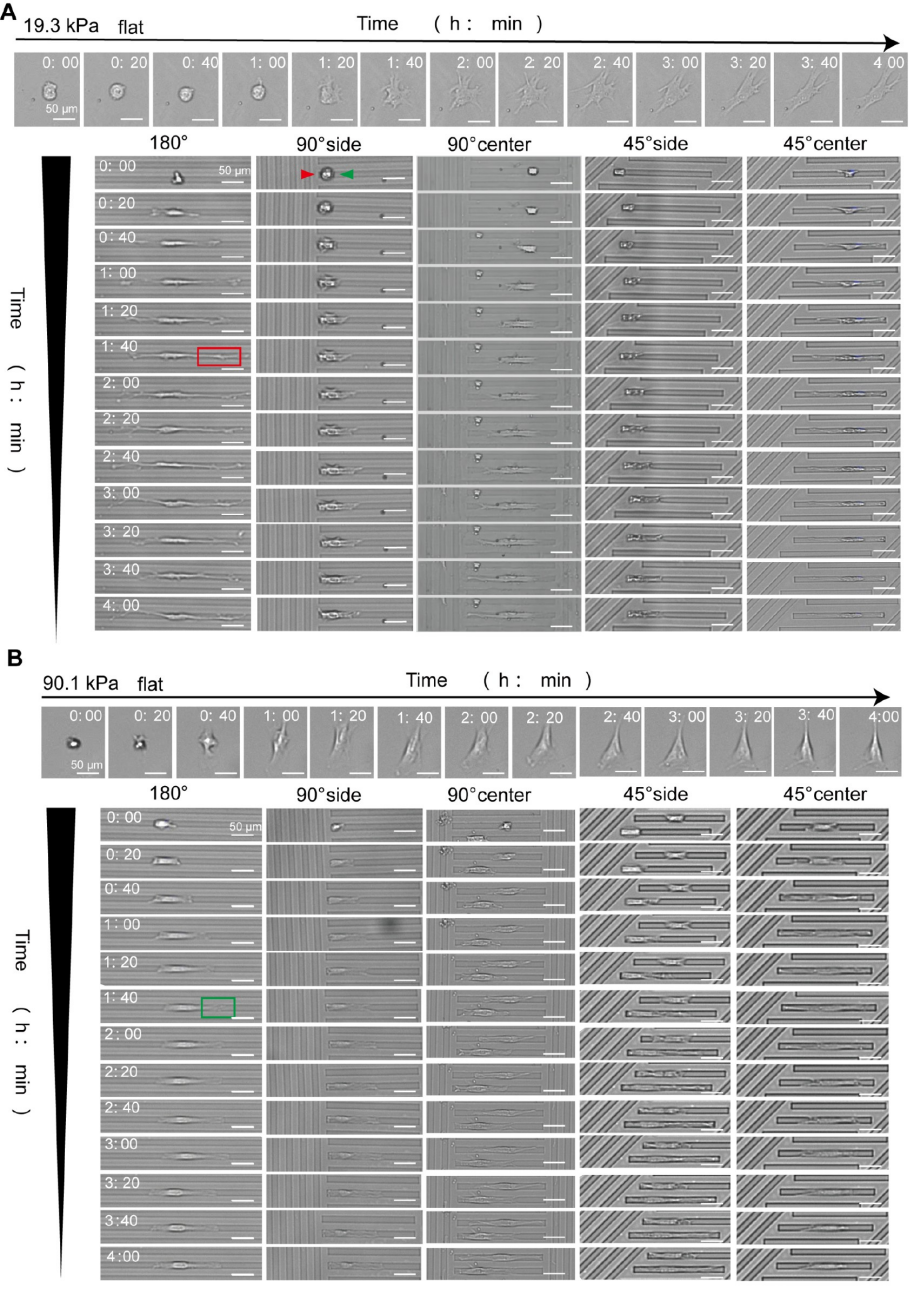
Figure 2 Morphology of HSF on stiffness-coupled topology substrates (A) Sequential morphological changes of cells on the 19.3 kPa substrate, captured at 20-min intervals for 4 h. (B) Morphological changes of cells on a 90.1 kPa substrate, documented at the same intervals.

Cells were seeded onto substrates and imaged using the Cytation 5 microscope. Previous observations indicated more pronounced cell changes during the first 4 h; hence, this study focused on the spreading pattern within this timeframe. In contrast to cells on flat substrates ( Figure 2A,B, flat), those on topologically varied substrates displayed width restriction, with 98% of HSF spreading within grooves. These cells grew inside and remained confined to the grooves. The cell morphology within the grooves is described here. The spreading pattern varied based on the initial adhesion location ( Figure 2). The number of cells in different substrate locations was quantified, and the results are presented as percentages in Table 3. On the 90° or 45° substrates, the cell locations were categorized into two classes. In one ( Figure 2A, 90° side), the cells adhered to one groove side, with the restricted end (indicated by a red arrow) limited in growth, while the free end (green arrow) spread freely. The other class ( Figure 2A, 90° center) featured cells adhering to the groove center and restricted at both ends during growth.

**Table:** **Table 3** Proportion of NC cells of different morphologies

Topological location	Ratio (19.3 kPa)	Ratio (90.1 kPa)
90° side	21.27%	34.78%
90° center	26.60%	26.08%
180° in 90° substrate	52.13%	39.13%
45° side	19.71%	34.06%
45° center	27.78%	12.08%
180° in 45° substrate	52.51%	53.84%

The cell spreading pattern exhibited considerable variation across different substrates. On the 19.3 kPa substrate, cells initially adhering to the 180° region ( Figure 2A, 180°) elongated at both ends along the groove. When stable, the cytosol was centrally located, resulting in a long, pike-shaped final morphology. Cells attached to the 90° side region ( Figure 2A, 90° side) experienced growth restriction on the confined end. In contrast, on the free side, they extended branches along the groove, culminating in an irregularly shaped rectangle at one end. The behavior of cells on the 45° side ( Figure 2A, 45° side) mirrored that on the 90° side. Cells attached to the 90° center ( Figure 2A, 90° center) were unrestricted in elongating along the grooves, stabilizing with the cytosol in a near-central position and having a long shuttle-like shape. Morphologically, cells at the 45° center ( Figure 2A, 45° center) resembled those at the 90° center. On the 90.1 kPa substrate, the overall process of cell spreading paralleled that on the 19.3 kPa substrate but with notable differences in the extent of branching at the cell ends. Cells on the 19.3 kPa substrate exhibited more elongated branches at both ends (highlighted in the red box in Figure 2A), whereas those on the 90.1 kPa substrate developed more robust branches (indicated in the green box in Figure 2A). This characteristic was consistent across the corresponding cell groups on both substrates, with cells on the 90.1 kPa substrate demonstrating more “fully”.

**Figure 3:**
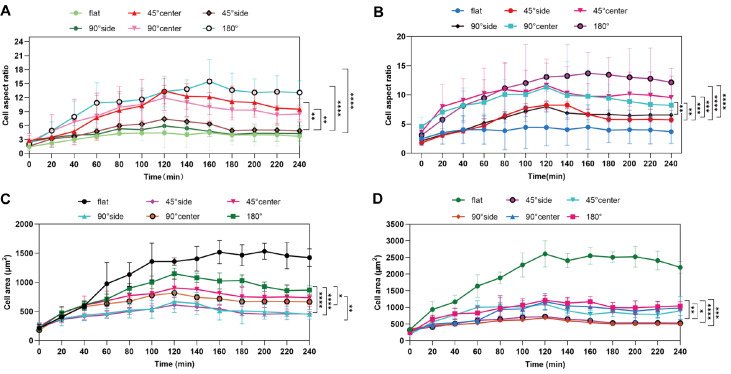
Figure 3 Aspect ratio and area statistics of HSF on stiffness-coupled topology substrates (A) Aspect ratio of cells on a 19.3 kPa substrate within 4 h. (B) Aspect ratio of cells on a 90.1 kPa substrate within the same timeframe. (C) Cell area on a 19.3 kPa substrate after 4 h. (D) Cell area on a 90.1 kPa substrate within the same period. Error bars represent the standard deviation. Data analysis was performed on 20 cells selected from each group using two-way ANOVA, with the experiment replicated three times.

To quantitatively characterize the changes in cell dynamics, the aspect ratios and areas of the cells ( Figure 3A–D) were statistically analyzed. With the exception of the 180° region, the general trend in cell spreading was similar: cells began spreading within 0–120 min, reaching peak aspect ratios and areas at approximately 120 min. Subsequently, the cells retracted from 120 min to 180 min, with aspect ratios and areas stabilizing without significant changes from 180 min to 240 min. In the 180° region, cells achieved maximum aspect ratios and areas at approximately 160 min, followed by slight retraction between 160 min and 240 min. Over the 0–4 h period and specifically at the 4 h mark, cells at 180° exhibited the largest aspect ratios and areas. This was followed by cells at the 45° and 90° centers, while those at the 45° and 90° sides had the smallest aspect ratios and areas. There was no statistically significant difference in the measurements of cells located at the same position on the 45° and 90° substrates ( *P*>0.05). Cells in the same topological location had larger areas and greater aspect ratios on the 90.1 kPa substrate ( *P*<0.01). These results indicate variations in cell spreading patterns based on the initial adhesion location. However, the overall trend of cell spreading within 4 h was consistent, with more pronounced spreading and polarization on the stiffer substrate.

### Influence of stiffness-coupled topological substrates on HSF differentiation

**Figure 4:**
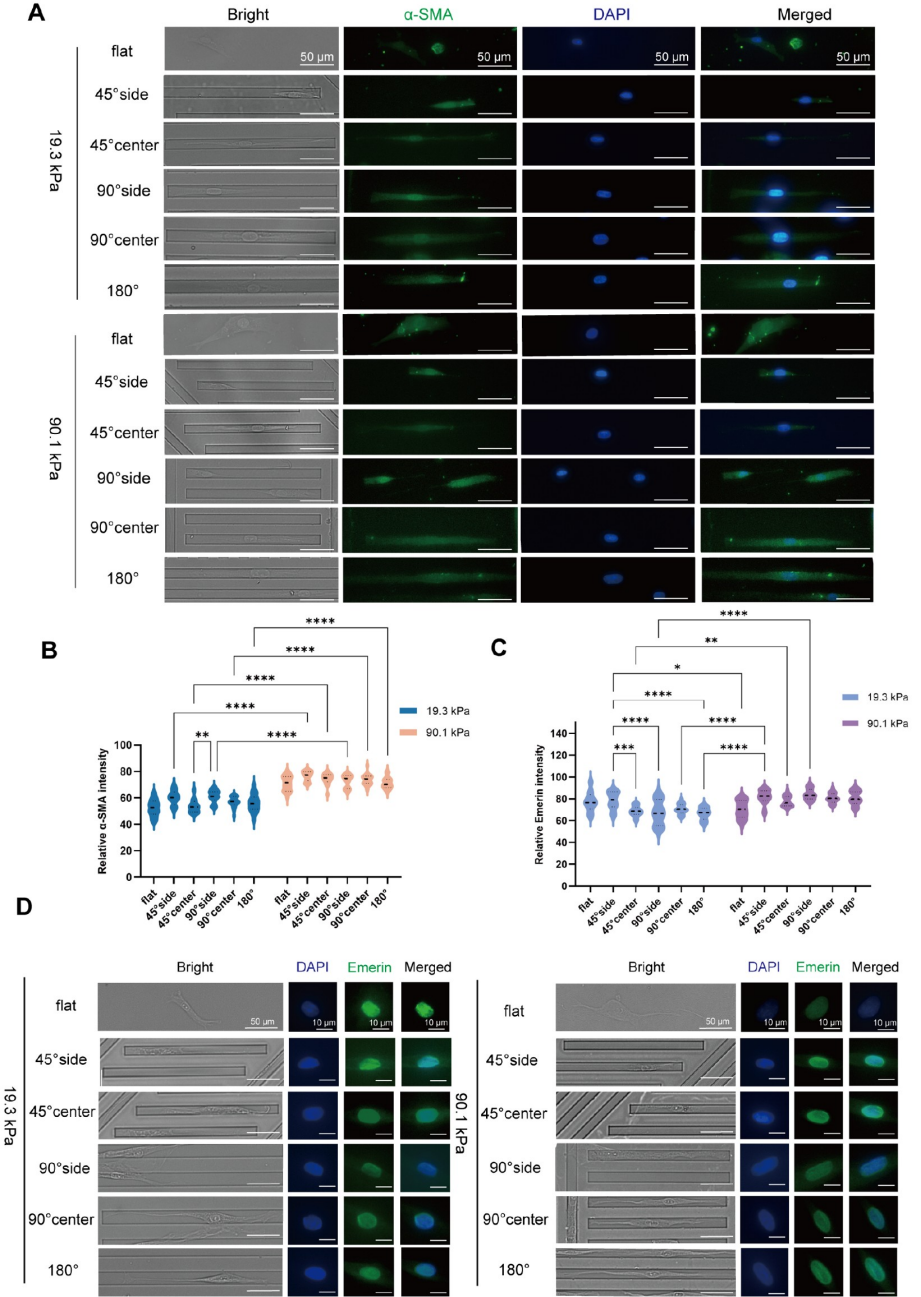
Figure 4 Differential expressions of α-SMA and Emerin after incubation of HSF with different stiffness-coupled topological substrates (A) Immunofluorescence staining of α-SMA showing nuclei (blue) and α-SMA (green). (B) Statistical analysis of the average fluorescence intensity of α-SMA. (C) Statistical analysis of the mean fluorescence intensity of Emerin. (D) Immunofluorescence staining of Emerin, depicting nuclei (blue), and Emerin (green). The error bars denote the standard deviation. Twenty cells from each group were analyzed using two-way ANOVA, and the experiment was repeated three times.

In HS tissue, fibroblasts tend to differentiate more into myofibroblasts [21], which secrete increased amounts of collagen, contributing to the heightened stiffness of the traumatized tissue. The morphology of cells cultured on different substrates varies significantly. However, the extent of fibrosis alteration when cell morphology stabilizes at 4 h remains unclear. To address this issue, α-smooth muscle actin (α-SMA), a key marker of HSF activation, was evaluated to assess the impact of substrate stiffness and topology on cellular fibrosis. Immunofluorescence staining revealed the distribution of α-SMA throughout the cell body, with a higher concentration occurring near the nucleus ( Figure 4A). On the 19.3 kPa substrate ( Figure 4B), cellular α-SMA expression was lowest on the flat substrate. α-SMA fluorescence intensity was greater in cells on the 90° side and 45° side than in those on the 180° side. The 90° central and 45° central areas exhibited the next highest intensities. There was no significant difference in α-SMA expression between cells at the same topological location of 45° and those at 90° ( *P*>0.05). On the 90.1 kPa substrate, the pattern of α-SMA expression in all regions mirrored that on the 19.3 kPa substrate, albeit with no significant intergroup differences ( *P*>0.05). However, α-SMA expression was significantly greater in cells on the 90.1 kPa substrate than in those on the 19.3 kPa substrate within the same topological areas ( *P*<0.0001). These findings suggest that the restriction of HSF stimulates its differentiation and that a stiffer substrate more effectively promotes this process.

### Siffness-coupled topological substrates have an effect on Emerin expression

Emerin, known for maintaining cellular homeostasis and mechanical signaling [22], was investigated in the context of the effects of an altered mechanistic microenvironment on cell morphology and differentiation. It remains to be determined whether Emerin, a nuclear membrane protein, plays a role in this process. To explore changes in Emerin, immunofluorescence staining was conducted. Emerin immunofluorescence images are shown in Figure 4D. The average fluorescence intensity of Emerin in various groups of cells was quantified, revealing that Emerin expression was lower in cells on the flat substrate at 90.1 kPa than in cells on the 19.3 kPa substrate and that cells on the 90.1 kPa substrate exhibited greater Emerin intensity than those on the 19.3 kPa substrate in comparable topological locations ( Figure 4C). Specifically, cells on the 19.3 kPa substrate showed increased Emerin expression in the 45° side and 90° side regions compared to the 180° area ( *P*<0.0001), with cells at the 45° center and 90° center displaying the next highest expression levels ( *P*<0.001). On the 90.1 kPa substrate, the trend of Emerin expression was similar to that on the 19.3 kPa substrate, yet there were no significant differences between the groups. These results suggest an increase in Emerin expression under cellular restriction and a greater average expression in the presence of stiffer substrates, indicating that Emerin is involved in the processes of cell spreading and differentiation.

### *Emerin* knockdown results in a new morphology in cells

**Figure 5:**
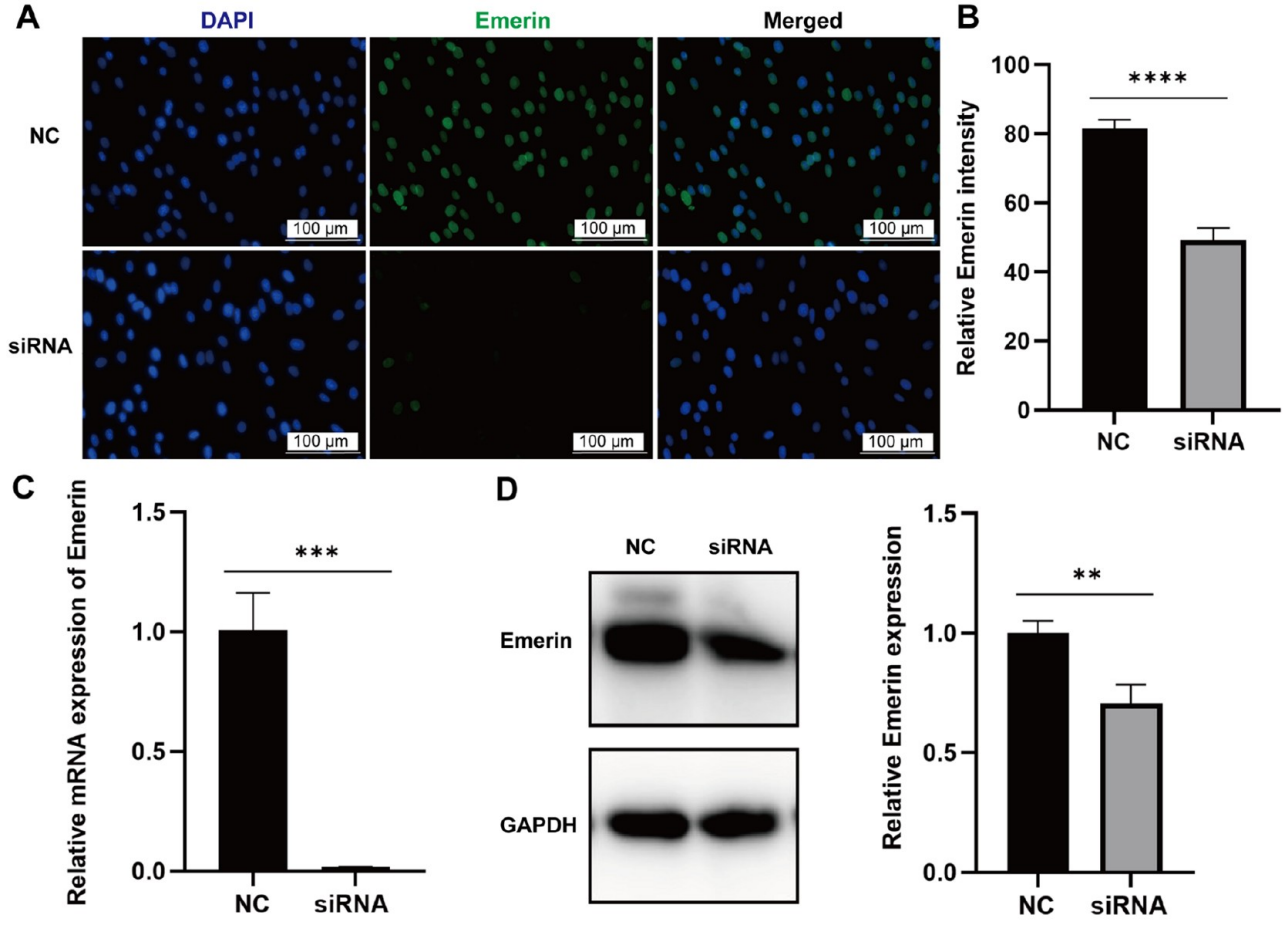
Figure 5 Transfection of HSF using RNAi technology to reduce Emerin expression (A) Immunofluorescence staining of HSF in both the negative control (NC) and siRNA groups. (B) Average immunofluorescence intensity of Emerin in the NC and siRNA groups, with 50 cells per group. (C) After Emerin siRNA transfection, mRNA expression levels were evaluated using qRT-PCR. (D) Following Emerin siRNA transfection, Emerin protein expression was assessed by western blot analysis. Data are presented as the mean±standard deviation ( n=3), and significance was tested using the t test.

To elucidate the role of Emerin in cellular processes, its expression was reduced using RNAi technology. Immunofluorescence staining revealed a marked difference in Emerin protein expression between the siRNA-treated group and the negative control (NC) group ( Figure 5B). mRNA expression in siRNA-transfected cells was approximately 98% lower than that in NC-transfected cells ( Figure 5C), and Emerin expression in the siRNA group was reduced to approximately 70% of that in the NC group ( Figure 5D), confirming successful *Emerin* knockdown.

Previous studies indicated no significant difference in cell growth between 45° and 90° substrates; thus, subsequent experiments focused on 90° and 180° substrates. Cells treated with siRNA were seeded onto these substrates, and morphological changes were observed after 4 h. The distribution of cells was categorized based on their initial adhesion location and spreading morphology, and the results are presented in Table 4. A greater proportion of siRNA-transfected cells initially adhered to the ridges (denoted as 90° ridges) than did NC-transfected cells. Hence, 90° ridges, 90° sides, and 90° centers were examined for the 90° substrate.

**Table:** **Table 4** Proportion of siRNA-transfected cells of different morpho-logies

Topological location	Ratio (19.3 kPa)	Ratio (90.1 kPa)
90° ridge	13.5%	10.51%
90° side spreading	15.31%	23.01%
90° side migration	16.90%	16.32%
90° center	19.72%	18.32%
180°	34.57%	31.84%

**Figure 6:**
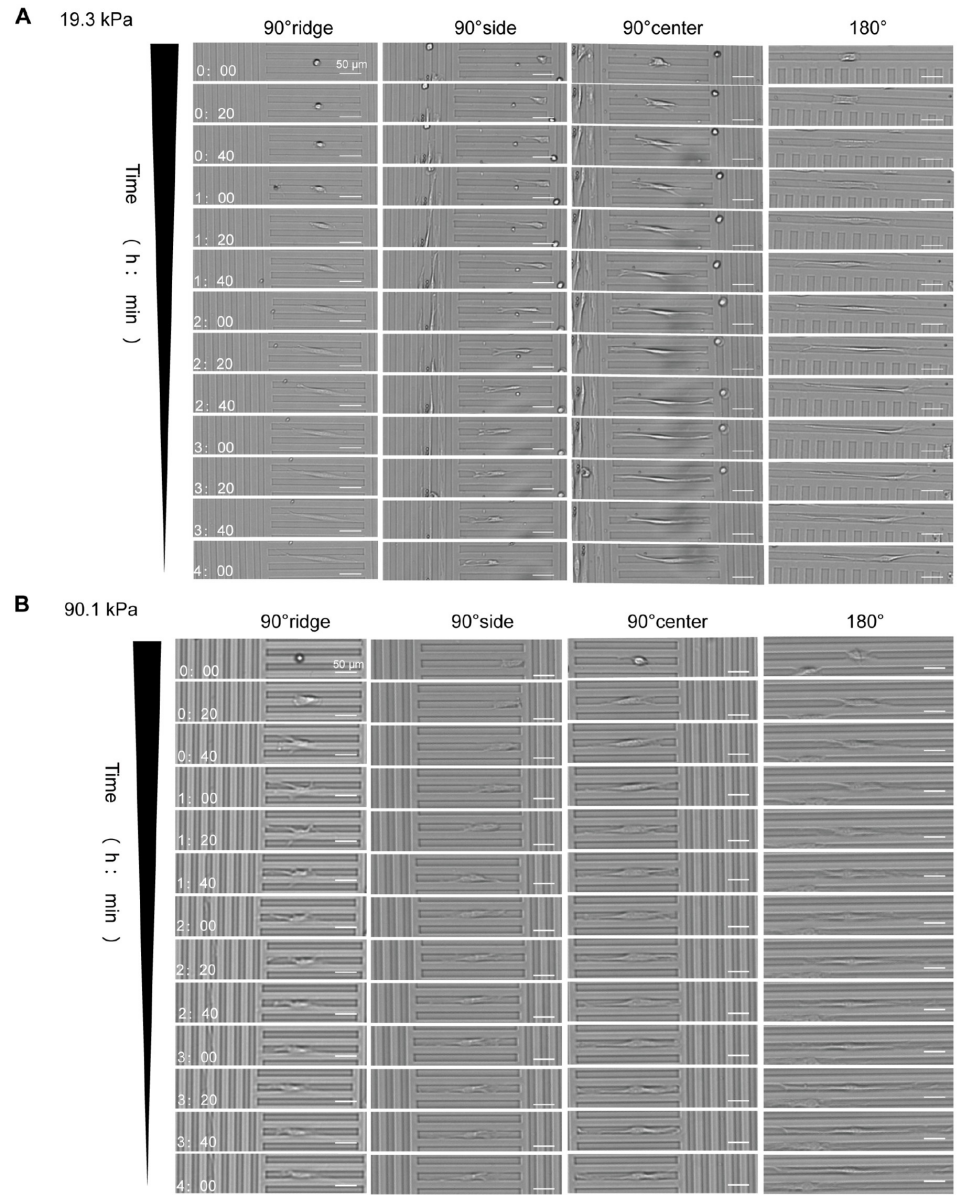
Figure 6 Morphology of siRNA group cells spread on topological substrates with different stiffnesses (A) Documented morphological changes of cells on a 19.3 kPa substrate, captured every 20 min for 4 h. (B) Similar documentation of morphological changes in cells on a 90.1 kPa substrate.

On the 19.3 kPa substrate, cells initially adhering to the ridges ( Figure 6A, 90° ridge) spread into elongated spike shapes along the ridges, with protrusions extending into the grooves, but the cytosol remained on the ridges. Cells attached to one side of the groove ( Figure 6A, 90° side) had their free ends elongate along the groove, leading to overall migration across the groove, with cells positioned on the opposite side at the 4 h mark. Cells initially adhering to the 90° groove center ( Figure 6A, 90° center) displayed an elongated cytosol that spread within the groove; their protrusions gradually extended and climbed onto adjacent ridges, resulting in a thin filamentous form within the groove at 4 h. Cells initially attached at 180° ( Figure 6A, 180°) elongated and spread along the grooves, with protrusions from one cell end climbing onto adjacent ridges.

On the 90.1 kPa substrate, cells initially adhering to the ridges ( Figure 6B, 90° ridge) spread initially along the ridges, with protrusions extending into adjacent grooves and the cytosol descending into and continuing to spread within these grooves. Cells initially attached to one side of the groove ( Figure 6B, 90° side) had their free ends elongate along the groove, resulting in overall cell migration toward the opposite side of the groove. At 4 h, the cellular cytosol was centrally located but had not reached the opposite groove side. The cells that initially adhered to the 90° center ( Figure 6B, 90° center) elongated within the grooves, with protrusions extending and spreading; at 4 h, stabilization was observed, with both poles restricted to the ends of the grooves. The cells that initially adhered at 180° ( Figure 6B, 180°) elongated and spread along the grooves, exhibiting greater protrusion lengths than did those at 90° at 4 h of spreading stabilization.

In summary, cells on the 19.3 kPa substrate were more elongated, with longer branches at the cell ends. Cells within the grooves, with cytosol located in these grooves, developed bipolar pseudopods that climbed onto the ridges. Cells spreading on ridges had their cytosol on the ridge, with pseudopods at both ends extending into the groove. On the 90.1 kPa substrate, the cellular cytosol appeared fuller, and cells in the grooves spread within these structures. Cells on the ridges initially spread along them before descending into adjacent grooves to elongate and spread.

### *Emerin* knockdown reduces HSF differentiation on substrates

**Figure 7:**
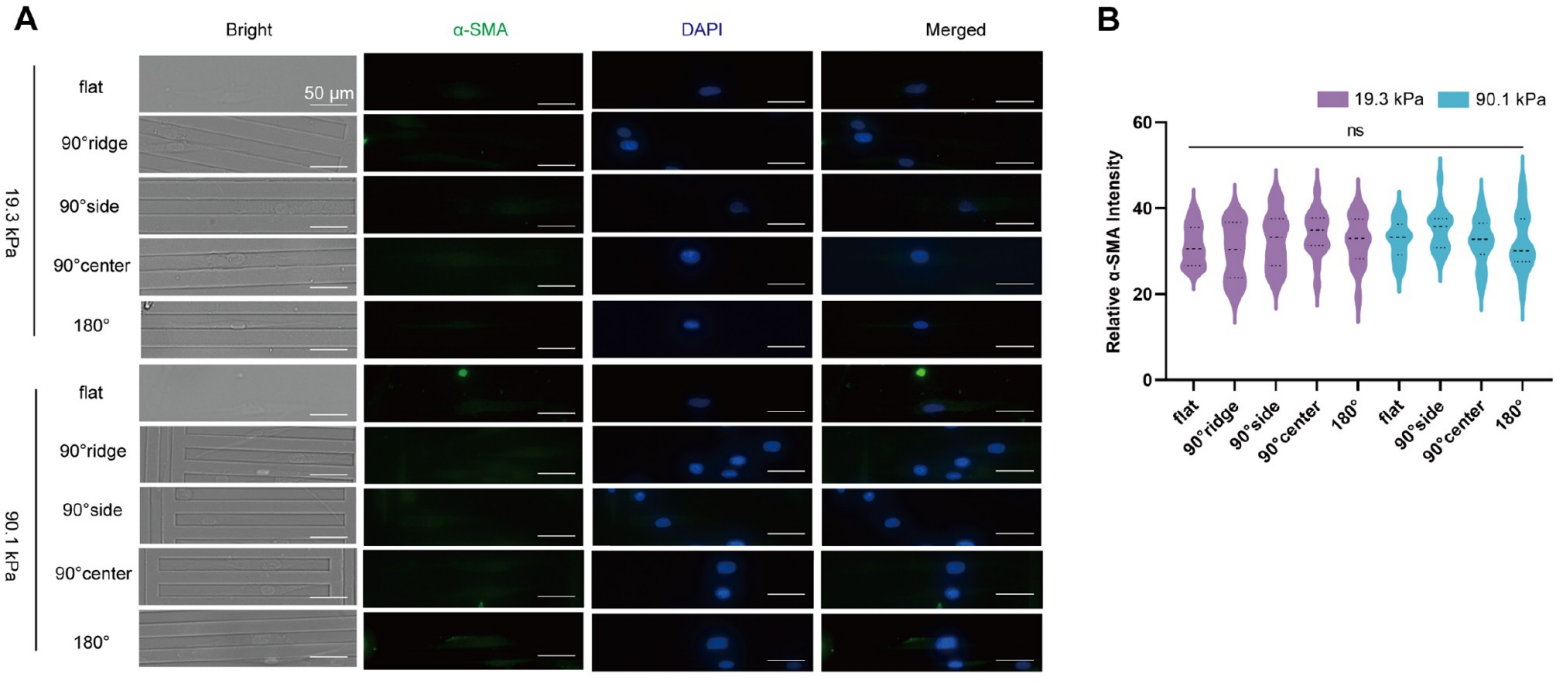
Figure 7 *Emerin* knockdown reduces α-SMA expression in cells (A) Immunofluorescence staining showing α-SMA (green) and nuclei (blue). (B) Statistical analysis of the average fluorescence intensity of α-SMA. The data from each group were analyzed using a t test, with 20 cells from each group selected for fluorescence intensity analysis.

Previous research indicated that silencing of the nuclear membrane protein nesprin2 gene inhibited mechanically induced myofibroblast differentiation [23]. This study aimed to determine whether *Emerin* knockdown similarly affects cell differentiation. After *Emerin* knockdown, the cells were seeded on hydrogel substrates, cultured for 4 h, fixed and stained. Based on the cell morphology shown in Figure 6 and the cell distribution in Table 4, cells on the 19.3 kPa substrate were selected for growth on the ridge (denoted as 90° ridge), the restricted area on one side of the groove (denoted as 90° side), the center of the groove (denoted as 90° center), and at 180°. Conversely, according to Figure 6B, at the 90° ridge, cells that initially grew on the ridge and subsequently descended into adjacent grooves were the focus for the 90.1 kPa substrate, primarily in the restricted region on one side of the groove (denoted as the 90° side), at the center of the groove (denoted as the 90° center), and at 180°. Immunofluorescence staining of α-SMA is shown in Figure 7A. Compared with that in the NC group, the α-SMA level in the siRNA group was significantly lower ( *P*<0.0001), but the difference between the siRNA groups was not significant ( Figure 7B). The results indicated a marked reduction in differentiation ability across all groups, with no significant difference observed between cells on the side, at 180°, or across both soft and hard substrates ( *P*>0.01). This finding suggested that the Emerin expression level is a key factor affecting differentiation following its knockdown.

### *Emerin* knockdown promotes HSF migration on the substrate

**Figure 8:**
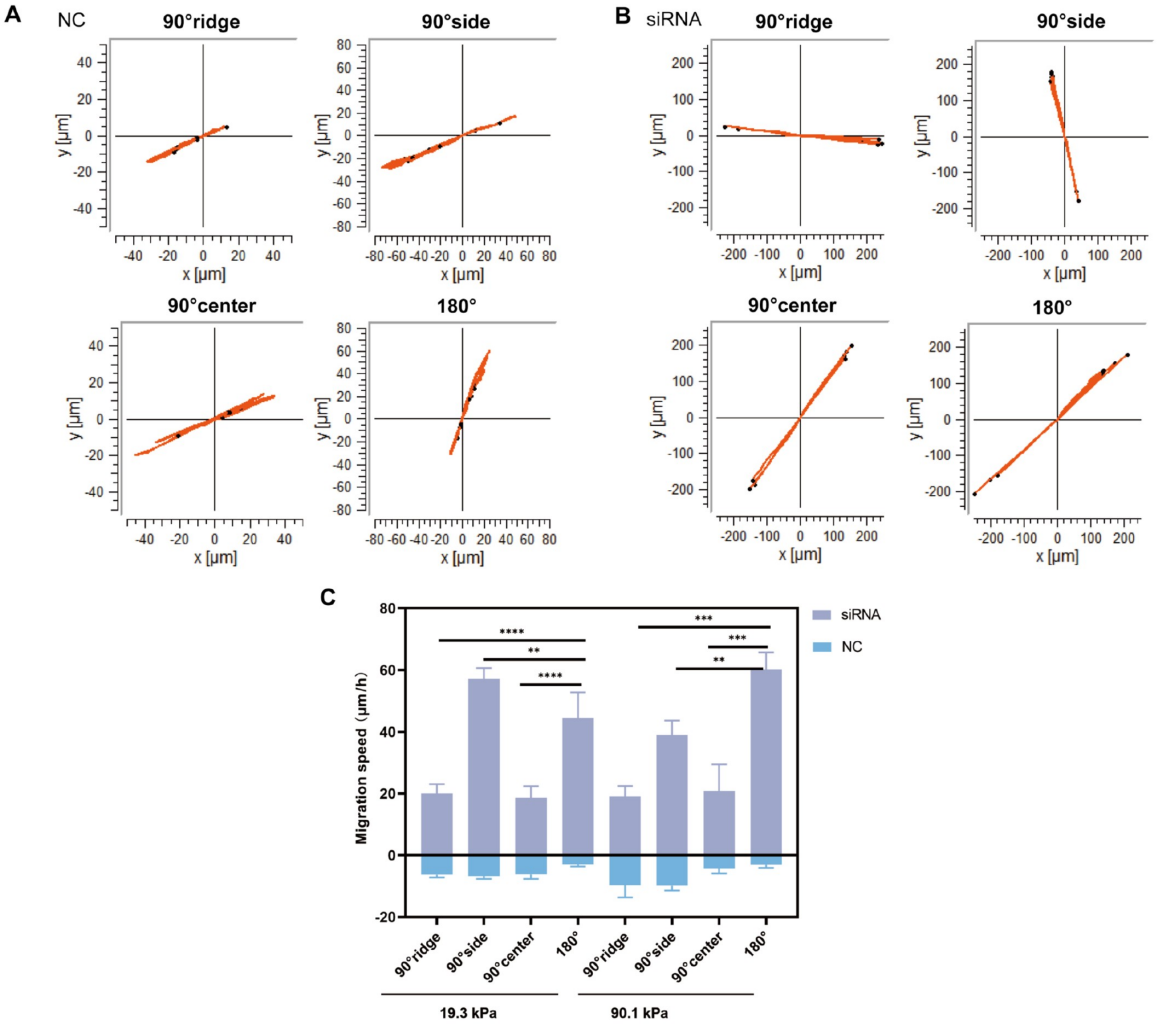
Figure 8 Knockdown of *Emerin* promotes cell migration (A) Migration paths of cells in the NC group were recorded using time-lapse microscopy at 4 h postinoculation. (B) Migration paths of cells in the siRNA group were similarly recorded. (C) Analysis of cell migration speed in both the NC and siRNA groups, with 8 cells chosen from each group. Data are expressed as the mean±standard error and were analyzed using one-way ANOVA.

In the study of cell morphology within the siRNA group, an increase in cell migration was observed. The distribution of cells with varying morphologies and migration patterns is detailed in Table 4. The migration tracks of NC cells ( Figure 8A) and siRNA-transfected cells ( Figure 8B) and their migration speeds ( Figure 8C) were analyzed. Compared to that of the NC group, the migration rate of the siRNA group cells was significantly greater. On the 19.3 kPa substrate ( Figure 8C), cells in the siRNA group exhibited increased migration speeds in the 90° side and 180° regions ( *P*<0.001), with 57.11±3.74 μm/h and 44.51±8.74 μm/h, respectively. Cells at the 90° ridge and 90° center migrated more slowly ( *P*<0.0001), with speeds of 20.03±3.21 μm/h and 18.61±4.03 μm/h, respectively. A similar trend was observed on the 90.1 kPa substrate. At 90°C, the cells on the 19.3 kPa substrate migrated faster at 57.11±3.74 μm/h than at 39.01±4.88 μm/h on the 90.1 kPa substrate ( *P*<0.0001). Conversely, cells in other locations on the 19.3 kPa substrate migrated more slowly than those on the 90.1 kPa substrate ( *P*<0.0001). These results indicated that cells in the siRNA group migrated faster on the 19.3 kPa substrate when restricted to one side, while the 90.1 kPa substrate facilitated migration to other locations. Decreased Emerin expression significantly enhances cell migration.

### *Emerin* knockdown decreases the cytoplasmic stiffness of HSF on the substrate

**Figure 9:**
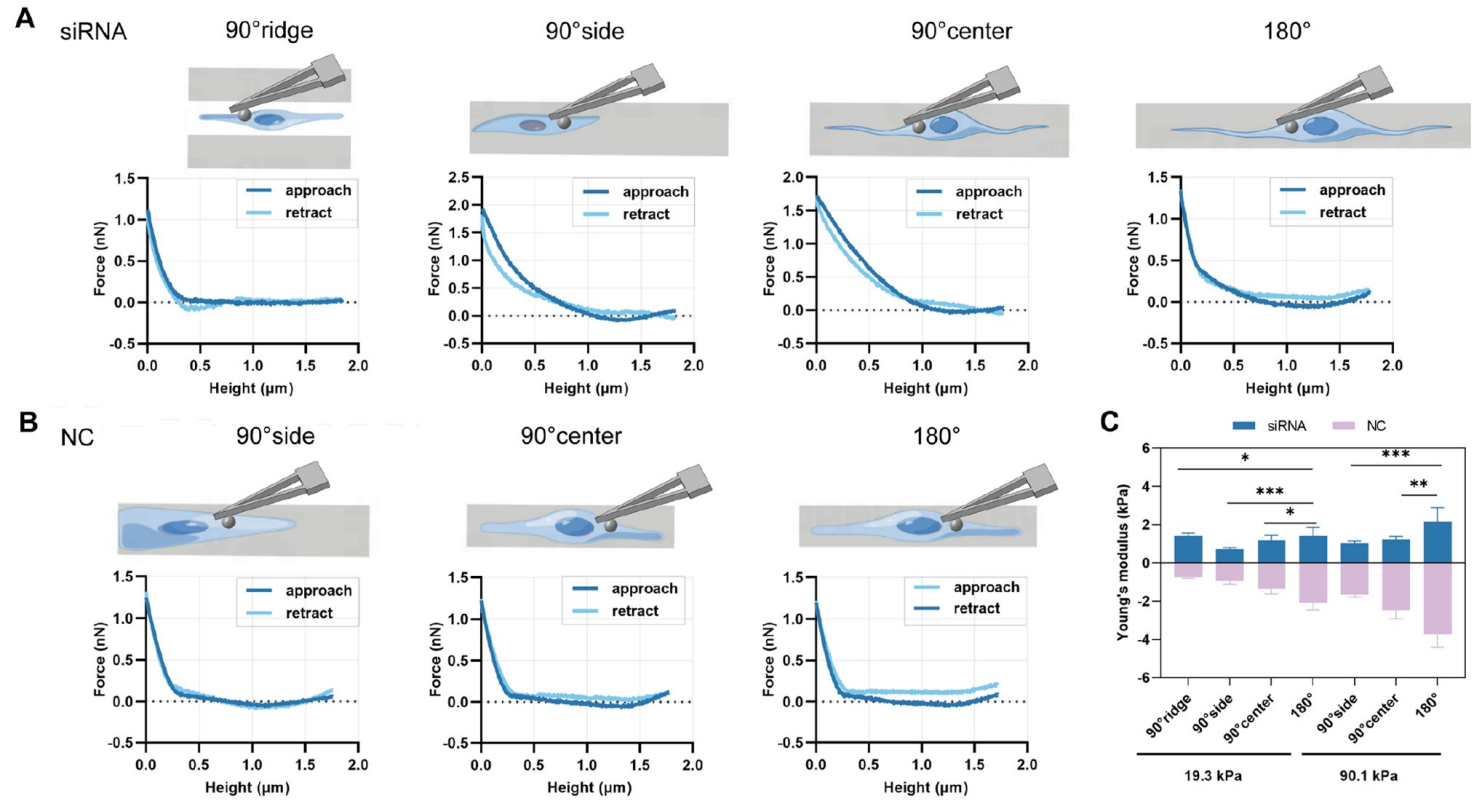
Figure 9 AFM indentation and mechanical properties of HSF cultured on different substrates (A) Force-distance curve of NC cells in a 19.3 kPa substrate, including cell measurement positions. (B) Force-distance curve of siRNA-transfected cells on a 19.3 kPa substrate, also showing measurement positions. (C) Young’s modulus statistics for cells in both the NC and siRNA groups. The data for each group were analyzed using two-way ANOVA and t tests.

Previous research has shown that cell migration typically coincides with changes in cellular stiffness [24]. Therefore, this study examined whether Emerin reduction-induced cell migration is also accompanied by alterations in cell stiffness. HSF exhibited typical elastic responses and stress relaxation ( Figure 9A,B), with probe measurement sites depicted in Figure 9A,B. The results indicated that the stiffness of cells in the siRNA group was significantly lower than that of NC cells ( Figure 9C). Both cell types demonstrated a similar pattern of stiffness change across substrates. Using the siRNA group as an example ( Figure 9C), the stiffness of cells on the 90.1 kPa substrate was markedly greater than that on the 19.3 kPa substrate. On the 19.3 kPa substrate, the cell stiffness was the lowest on the 90° side (0.70±0.09 kPa) and highest in the 90° ridge and 180° regions (1.40±0.13 kPa and 1.40±0.44 kPa, respectively). On the 90.1 kPa substrate, the stiffness was the lowest on the 90° side (1.03±0.10 kPa) and highest in the 180° region (2.15±0.73 kPa). These data suggest that increased cell restriction leads to reduced cell stiffness and that decreased Emerin expression also decreases cell stiffness.

## Discussion

Dermatofibrosis, a physiologic condition stemming from abnormal skin cell repair [25], not only impacts patient appearance but also, in severe cases, can hinder limb mobility due to scar contracture [26]. This condition places a significant burden on both the physical and mental health of patients. A key aspect of fibrosis is the hyperdifferentiation of skin fibroblasts into myofibroblasts. These overdifferentiated cells produce excessive collagen, leading to the hardening of the extracellular matrix and a more disorganized collagen fiber arrangement. This abnormal physiological environment in turn affects fibroblasts, causing them to respond to mechanical stress, regulate protein expression, and modify the extracellular matrix environment in response to mechanical stimuli. In wound healing, the migratory capacity of myofibroblasts is crucial for the contraction and maturation of granulation tissue, and it is also a significant factor in scar tissue contracture deformity posthealing [27].

In recent years, extensive reports on the regulatory relationship between nuclear skeleton proteins and cell behavior have been published [ 28, 29] . However, the role of nuclear backbone proteins in the phenotypic transformation of HSF within the matrix microenvironment remains unclear. This study aimed to replicate the three-dimensional structure of collagen fiber bundles in skin wounds and scar tissue *in vitro* by constructing a substrate culture system with varying stiffness, cross-groove topology, and stiffness-cross-groove coupling. This research focused on investigating how substrate mechanical stimulation regulates HSF phenotypic transformation and exploring the mechanism of aberrant differentiation of nucleoskeletal proteins in the fibroblast microenvironment. Stiffness primarily influences the cell area and aspect ratio, while topology predominantly affects cell morphology. On stiffness-coupled topological substrates, Emerin modulates cell differentiation and migration rates by adjusting stiffness, presenting a potential strategy to combat HS development.

In this study, PAA substrates with two stiffness levels, 19.3 kPa and 90.1 kPa, were prepared to approximate the elastic moduli of normal traumatized skin tissue and scar tissue. Topological grooves crossed at 90° and 45° were designed to simulate the physical morphology of scarred collagen fiber bundles, constructing stiffness-coupled topological structures for an *in vitro* microenvironmental culture system.

The morphology, area, and aspect ratio of fibroblasts accurately reflect cellular responses in both normal skin and scar tissue. Our findings indicate that a harder substrate facilitates fibroblast spreading and elongation, highlighting substrate stiffness as a crucial determinant of cell growth ( Figure 3). The topology primarily influences cell morphology, particularly when growth is significantly restricted on one side of the cell ( Figure 2). Reducing Emerin expression in basal cells results in abnormal distribution and growth preference toward higher “ridges”, possibly due to cell softening. Additionally, the morphology of cells, especially those severely restricted on one side, is influenced by changes in cell stiffness and migration behavior, differing markedly from those of the control group.

α-SMA expression is elevated on harder substrates, suggesting that more restrictive and stiffer substrates promote cell spreading and fibrosis, which is consistent with existing research [30]. Variations in Emerin expression across different suprabasal cell differentiation states indicate its potential involvement in HSF differentiation. A decrease in Emerin corresponds to reduced cellular differentiation, and no significant difference was observed in the degree of cellular fibrosis across various topologies. This finding underscores the role of Emerin in cell differentiation and its positive correlation with α-SMA expression. During the proliferative and regressive phases of scarring, α-SMA level directly impacts the extent of scar tissue fibrosis [ 31– 33] . Myofibroblasts contribute to scar tissue formation, thickening, contraction, and stiffness through increased α-SMA expression, stress fiber formation, excessive collagen synthesis, secretion, and remodeling of the extracellular matrix (ECM) [ 34, 35] . This study suggested that inhibiting Emerin expression could effectively reduce myofibroblast differentiation, suggesting a potential avenue for improving hypertrophic scarring treatment.

In the proliferative and reparative stages of wound healing, matrix components transform into granulation tissue elements to cover the wound. α-SMA exhibits strong contractile properties at this stage [36], and myofibroblast differentiation is beneficial for granulation tissue maturation. Cell restriction increases fibroblast-to-myofibroblast differentiation; thus, promoting cell restriction through external forces or changes in the extracellular matrix environment can enhance the rapid repair of granulation tissue.

Interestingly, when *Emerin* was knocked down, the cells exhibited pronounced migratory behavior. We quantified the migration of control and siRNA-treated cells. The results indicated that *Emerin* knockdown significantly enhanced the migration rate of cells, with the harder 90.1 kPa substrate more effectively facilitating cell migration, consistent with existing research [37]. However, an anomaly was observed: cells in the siRNA group within the more restricted region (90° side) migrated faster on the 19.3 kPa substrate than on the 90.1 kPa substrate. We hypothesize that this cell behavior might represent a self-protective adaptation to the restricted environment, effectively “escaping” from confined areas. Numerous studies have shown that cell migration is closely linked to cellular stiffness; furthermore, to confirm whether changes in nuclear membrane proteins influence cell stiffness, this study measured the elastic modulus of cells. The findings revealed that higher cell migration rates correspond to lower elastic moduli, particularly in regions with restricted cell growth. After Emerin reduction, the cells appeared to escape the obstructed regions by softening, thereby facilitating cytosolic migration, which was especially evident at 19.3 kPa, where cell migration rates significantly increased. During the initial and intermediate phases of skin wound healing, a critical factor influencing healing is the efficient and rapid migration of cells to the wound center [38]. The results of this study demonstrated that reduced Emerin expression in the nucleus softens cells and increases their migration rate, promoting more cells to migrate to the wound site and accelerating granulation tissue repair. The promotion of myofibroblast migration by *Emerin* knockdown is thus a crucial aspect of its antiscarring effect.

Our study proposes a novel conclusion: cell-restricted enhancement of Emerin expression under stiffness-coupled topological substrates promotes cellular fibrosis. Decreasing Emerin expression not only attenuates α-SMA expression but also promotes migration by “softening” cells. This finding offers insights for initial wound healing and for treating keloid hyperplasia during regression. This study revealed the role of Emerin in regulating fibroblast phenotypic transformation and cell migration, validating the responses of HSF to the mechanical environment of the extracellular matrix. However, there are several limitations to overcome in this study. For example, the expression of another LEM domain protein (Lap2, Man1) as a control will be a useful addition, and the specific molecular mechanisms by which nuclear membrane proteins regulate cellular behavior remain unclear. In the future, we will further explore the mechanism of HSF perception of mechanical signals and the role of the nucleus in this process to provide a reference for understanding the formation and prevention of scar fibrotic lesions.

## References

[1] Grabowski G, Pacana MJ, Chen E (2020). Keloid and hypertrophic scar formation, prevention, and management: standard review of abnormal scarring in orthopaedic surgery. J Am Acad Orthop Surg.

[2] Goffin JM, Pittet P, Csucs G, Lussi JW, Meister JJ, Hinz B (2006). Focal adhesion size controls tension-dependent recruitment of α-smooth muscle actin to stress fibers. J Cell Biol.

[3] Macarak EJ, Wermuth PJ, Rosenbloom J, Uitto J (2021). Keloid disorder: fibroblast differentiation and gene expression profile in fibrotic skin diseases. Exp Dermatol.

[4] Da Costa V, Wei R, Lim R, Sun CH, Brown JJ, Wong BJF (2008). Nondestructive imaging of live human keloid and facial tissue using multiphoton microscopy. Arch Facial Plast Surg.

[5] Johnson LA, Rodansky ES, Sauder KL, Horowitz JC, Mih JD, Tschumperlin DJ, Higgins PD (2013). Matrix stiffness corresponding to strictured bowel induces a fibrogenic response in human colonic fibroblasts. Inflamm Bowel Dis.

[6] Raab M, Swift J, Dingal PCDP, Shah P, Shin JW, Discher DE (2012). Crawling from soft to stiff matrix polarizes the cytoskeleton and phosphoregulates myosin-II heavy chain. J Cell Biol.

[7] Liu Z, Mo H, Liu R, Niu Y, Chen T, Xu Q, Tu K (2021). Matrix stiffness modulates hepatic stellate cell activation into tumor-promoting myofibroblasts via E2F3-dependent signaling and regulates malignant progression. Cell Death Dis.

[8] Ishimoto T, Kawata K, Sakai T, Yoshikawa H, Nakano T (2012). Regeneration of bone mass and bone quality around implant with grooves for aligning bone cells in rabbit hindlimb bones. Materials Science Forum.

[9] Ghrebi S, Hamilton DW, Douglas Waterfield J, Brunette DM (2013). The effect of surface topography on cell shape and early ERK1/2 signaling in macrophages; linkage with FAK and Src. J BioMed Mater Res.

[10] Yom-Tov O, Seliktar D, Bianco-Peled H (2014). Cell morphology in injectable nanostructured biosynthetic hydrogels. J Biomed Mater Res B Appl BioMater.

[11] Su WT, Liao YF, Lin CY, Li LT (2010). Micropillar substrate influences the cellular attachment and laminin expression. J BioMed Mater Res.

[12] Lammerding J, Hsiao J, Schulze PC, Kozlov S, Stewart CL, Lee RT (2005). Abnormal nuclear shape and impaired mechanotransduction in emerin-deficient cells. J Cell Biol.

[13] Nastały P, Purushothaman D, Marchesi S, Poli A, Lendenmann T, Kidiyoor GR, Beznoussenko GV (2020). Role of the nuclear membrane protein Emerin in front-rear polarity of the nucleus. Nat Commun.

[14] Du Z, Zhu T, Lin M, Bao Y, Qiao J, Lv G, Xie Y (2022). A novel mutation in human
*EMD* gene and mitochondrial dysfunction in emerin knockdown cardiomyocytes. J Cell Mol Medi.

[15] Essawy N, Samson C, Petitalot A, Moog S, Bigot A, Herrada I, Marcelot A (2019). An emerin LEM-domain mutation impairs cell response to mechanical stress. Cells.

[16] Willer MK, Carroll CW (2017). Substrate stiffness-dependent regulation of the SRF-Mkl1 co-activator complex requires the inner nuclear membrane protein Emerin. J Cell Sci.

[17] Lü D, Luo C, Zhang C, Li Z, Long M (2014). Differential regulation of morphology and stemness of mouse embryonic stem cells by substrate stiffness and topography. Biomaterials.

[18] Luo C, Lü D, Zheng L, Zhang F, Zhang X, Lü S, Zhang C (2021). Hepatic differentiation of human embryonic stem cells by coupling substrate stiffness and microtopography. Biomater Sci.

[19] Li Z, Gong Y, Sun S, Du Y, Lü D, Liu X, Long M (2013). Differential regulation of stiffness, topography, and dimension of substrates in rat mesenchymal stem cells. Biomaterials.

[20] Friedrich EE, Niknam‐Bienia S, Xie P, Jia SX, Hong SJ, Mustoe TA, Galiano RD (2017). Thermal injury model in the rabbit ear with quantifiable burn progression and hypertrophic scar. Wound Repair Regen.

[21] Crawford J, Nygard K, Gan BS, O′Gorman DB (2015). Periostin induces fibroblast proliferation and myofibroblast persistence in hypertrophic scarring. Exp Dermatol.

[22] Snyers L, Löhnert R, Weipoltshammer K, Schöfer C, Discher D (2022). Emerin prevents BAF-mediated aggregation of lamin A on chromosomes in telophase to allow nuclear membrane expansion and nuclear lamina formation. Mol Biol Cell.

[23] Xu Q, Miao Y, Ren J, Sun Y, Li C, Cai X, Wang Z (2022). Silencing of Nesprin‐2 inhibits the differentiation of myofibroblasts from fibroblasts induced by mechanical stretch. Int Wound J.

[24] Kita K, Asanuma K, Okamoto T, Kawamoto E, Nakamura K, Hagi T, Nakamura T (2021). Cytoskeletal actin structure in osteosarcoma cells determines metastatic phenotype via regulating cell stiffness, migration, and transmigration. Curr Issues Mol Biol.

[25] Insights into the Pathophysiology of Hypertrophic Scars and Keloids: How Do They Differ?
*
Adv Skin Wound Care
* 2018, 31: E1. https://doi.org/10.1097/01.ASW.0000527945.80449.45.

[26] Honardoust D, Ding J, Varkey M, Shankowsky HA, Tredget EE (2012). Deep dermal fibroblasts refractory to migration and decorin-induced apoptosis contribute to hypertrophic scarring. J Burn Care Res.

[27] Darby IA, Laverdet B, Bonté F, Desmoulière A (2014). Fibroblasts and myofibroblasts in wound healing. CCID.

[28] Chen L, Jiang F, Qiao Y, Li H, Wei Z, Huang T, Lan J (2018). Nucleoskeletal stiffness regulates stem cell migration and differentiation through lamin A/C. J Cell Physiol.

[29] Lavenus SB, Vosatka KW, Caruso AP, Ullo MF, Khan A, Logue JS (2022). Emerin regulation of nuclear stiffness is required for fast amoeboid migration in confined environments. J Cell Sci.

[30] Li Y, Xiao Z, Zhou Y, Zheng S, An Y, Huang W, He H (2019). Controlling the multiscale network structure of fibers to stimulate wound matrix rebuilding by fibroblast differentiation. ACS Appl Mater Interfaces.

[31] Hu X, Wang H, Liu J, Fang X, Tao K, Wang Y, Li N (2013). The role of ERK and JNK signaling in connective tissue growth factor induced extracellular matrix protein production and scar formation. Arch Dermatol Res.

[32] Suleiman M, Singh R, Stewart C (2007). Apoptosis and the cardiac action of insulin-like growth factor I. Pharmacol Ther.

[33] Javelaud D, Mauviel A (2004). Mammalian transforming growth factor-βs: Smad signaling and physio-pathological roles. Int J Biochem Cell Biol.

[34] Stoica AE, Grumezescu AM, Hermenean AO, Andronescu E, Vasile BS (2020). Scar-free healing: current concepts and future perspectives. Nanomaterials.

[35] Monavarian M, Kader S, Moeinzadeh S, Jabbari E (2019). Regenerative scar-free skin wound healing. Tissue Eng Part B Rev.

[36] Zhong L, Li M, Fu X (2020). Biological approaches for hypertrophic scars. Int Wound J.

[37] Wu KY, Xie H, Zhang ZL, Li ZX, Shi L, Zhou W, Zeng J (2022). Emerin knockdown induces the migration and invasion of hepatocellular carcinoma cells by up-regulating the cytoplasmic p21. Neoplasma.

[38] Leng M, Peng Y, Wang H. Research advances on the biomechanical micro- environment facilitated wound repair through the regulation of cell migration.
*
Zhonghua Shao Shang Za Zhi
* 2022, 38: 90–94. https://doi.org/10.3760/cma.j.cn501120-20200921-00419.

